# Stigma associated with leprosy among patients, contacts, and the general population in an endemic region of Brazil^☆^

**DOI:** 10.1016/j.abd.2026.501301

**Published:** 2026-03-09

**Authors:** Patrícia Tavares Cruz, Hélio Amante Miot, Carolina Talhari, Valderiza Lourenço Pedrosa, Raquel da Mata Serique, Amanda Gabrielle dos Santos Cordeiro, Jaqueline da Silva Mendes, Susan Smith Doria, Jorge Ewerton dos Santos Sales, Alexandra de Freitas Costa, Sinésio Talhari

**Affiliations:** a Department of Teaching and Research, Fundação Hospitalar de Dermatologia Tropical e Venereologia Alfredo da Matta, Manaus, AM, Brazil; b Department of Infectology, Dermatology, Imaging Diagnosis, and Radiotherapy, Faculty of Medicine, Universidade Estadual Paulista, Botucatu, SP, Brazil

**Keywords:** Leprosy, Neglected diseases, Psychosocial impact, Public health, Social stigma

## Abstract

**Background:**

Leprosy is associated with social exclusion and discrimination. Historical records of compulsory isolation, severe clinical manifestations, and fear of contagion have contributed to the perpetuation of prejudice and discriminatory attitudes. In Brazil, the psychosocial impact of leprosy among patients, contacts, and the general population remains poorly understood.

**Objective:**

To assess the stigma associated with leprosy and to identify factors influencing its impact among patients, contacts, and the General Population (GP).

**Methods:**

A cross-sectional study was conducted, including three groups (patients, contacts, and GPs) from the metropolitan region of Manaus – AM (Brazil). Demographic and clinical data were collected, and stigma was evaluated using the Explanatory Model Interview Catalogue-Affected People (EMIC-AP) for patients and the EMIC-Community Stigma Scale (EMIC-CSS) for contacts and GPs. The impact of different aspects was assessed within each group, and clinicodemographic factors associated with higher stigma were explored.

**Results:**

A total of 214 patients, 104 contacts, and 393 GPs were evaluated. The EMIC-AP mean (SD) score among patients was 16 (9), whereas the EMIC-CSS scores were 15 (7) for contacts and 12 (6) for the GP group. Network analysis revealed that feelings of shame, negative self-opinion, and social avoidance were central elements of stigma across groups. Female sex among patients (β = -2.9; p = 0.03), higher education among contacts (β = 3.2; p = 0.03); and younger age and religious practice among the GPs (β = -1.3 and 1.3; p < 0.05) were associated with higher stigma scores. After standardization, contacts and GP exhibited greater stigma perception than patients (θ = 0.02 and -0.06 vs. -0.26; p < 0.05).

**Study Limitations:**

Non-randomized sample from a single center.

**Conclusions:**

Leprosy-related stigma persists across patients, contacts, and GPs, reflecting deep-rooted social and cultural misconceptions. Beyond medical cure, achieving the “Zero Leprosy” goals demands integrated actions that promote education, community engagement, and psychosocial support to reduce prejudice and promote social inclusion.

## Introduction

Leprosy is one of the oldest chronic infectious diseases known to humankind, caused by *Mycobacterium leprae* and *M. lepromatosis*.^1^ It is characterized by localized or disseminated skin lesions and involvement of peripheral nerves, which may lead to sensory, motor, and autonomic loss, resulting in physical disabilities, deformities, and psychosocial suffering.^2^, ^3^ These manifestations are responsible for the strong social stigma historically associated with the disease, often reinforced by religious interpretations and compulsory isolation in leprosaria.^4^, ^5^ Despite advances in diagnosis and curative treatment, social discrimination related to leprosy persists, even among patients and their families, negatively affecting treatment adherence, self-esteem, and social integration.^1^, ^6^

Despite the global decline in prevalence, Brazil remains among the countries with the highest incidence rates, and leprosy continues to be an important cause of physical disability in the country.^7^ Early diagnosis, active surveillance, and appropriate treatment are essential to interrupt transmission chains and prevent sequelae.^3^, ^8^, ^9^

Systematic measurement of stigma through validated instruments is essential to support public policies aimed at reducing social and cultural barriers to early diagnosis and comprehensive care for affected individuals. The persistence of stigma, even in the presence of effective and widely available treatment, justifies investigating its perception among different social groups.^10^ Understanding how prejudice and social exclusion are experienced by some patients can guide strategies that promote not only a cure but also their social integration.^11^, ^12^

The introduction of multidrug therapy in 1981 revolutionized leprosy treatment, replacing the single use of dapsone for life and ending the paradigm that the disease was incurable. The combination of dapsone, rifampicin, and clofazimine proved highly effective and had a low risk of inducing resistance, allowing the cure of approximately 15 million people and a marked reduction in global prevalence.^9^, ^13^ Following this success, the World Health Organization (WHO) set, in 1991, the goal of eliminating leprosy as a public health problem by the year 2000, later postponed to 2005 due to the persistence of the disease in endemic countries such as Brazil, India, and Indonesia. Since 2016, the WHO has incorporated stigma reduction as a strategic axis of its global actions. The Global Leprosy Strategy 2021–2030 aims to achieve elimination of the disease through the “zero leprosy” targets: zero cases, zero disability, and zero stigma.^13^

The social stigma linked to leprosy, aggravated by a lack of understanding about the disease and the erroneous belief that it is rare, hampers disease control, making patients more vulnerable to discrimination and exclusion. Moreover, social marginalization combined with unfavorable socioeconomic conditions contributes to the persistence of leprosy and worsens its consequences. Effective treatment and elimination of physical disabilities (and deformities) associated with the disease can significantly reduce stigma, fostering public perception that leprosy is both treatable and curable.^14^, ^15^

Quantitative assessment of leprosy-related stigma is recommended by the Brazilian Ministry of Health and is performed using two instruments: The Explanatory Model Interview Catalogue – Affected People (EMIC-AP) and the Explanatory Model Interview Catalogue – Community Stigma Scale (EMIC-CSS).^16^, ^17^ The EMIC-AP is widely used and internationally validated, including in Brazil, where the adapted version enables assessment of perceived stigma among people affected by leprosy. It consists of 15 items with Likert-type responses and scores ranging from zero to 45, where higher scores indicate greater stigma. The EMIC-CSS, in turn, is applied to the community and contacts to assess social attitudes toward people affected by leprosy. It also includes 15-questions, with a maximum score of 30-points, designed to identify negative community attitudes toward those affected. Both scales, validated across multiple cultures and recently in Brazil, are valuable tools for investigating stigma and generating data to inform social and public health interventions.^18^

Even after decades of public policies targeting eradication, prejudice persists at different levels of society, reflecting a historical legacy of exclusion and misinformation. Evaluating the magnitude and determinants of this stigma is essential to understanding its contemporary manifestations and to guide intersectoral strategies that go beyond the biomedical perspective. Therefore, this study aims to investigate the extent of stigma associated with leprosy among patients, contacts, and the general population not related to the disease (GP), as well as to identify factors contributing to a greater perception of stigma in the state of Amazonas, an endemic region of Brazil.

## Methods

### Design of the study

This was a cross-sectional, non-randomized study conducted at the Fundação Hospitalar Alfredo da Matta (FUHAM), Manaus – AM (Brazil), reference center between May 2024 and August 2025.

### Ethical aspects

The study was approved by the Research Ethics Committee of FUHAM, Manaus – AM (Brazil) under protocol number 79502724.1.0000.0002. All participants were interviewed after reading and signing the Informed Consent Form, in accordance with Good Clinical Practices (Document of the Americas) and with full respect for human dignity.

### Population of the study

Adults aged 18-years or older, of both genders, residing in the metropolitan region of Manaus – AM (Brazil), who agreed to participate in the study, were included.

The study population was divided into three groups: 1) Patients: Individuals diagnosed with leprosy, confirmed according to the Clinical Protocol and Therapeutic Guidelines for Leprosy, including new or ongoing cases, with or without physical disability, attended and under treatment in two reference services (FUHAM and leprosarium of the Colônia Antonio Aleixo – Manaus – AM - Brazil). 2) Contacts: Individuals accompanying those patients during consultations who had lived with them for at least five years. 3) GP: Individuals without a family history of leprosy and not employed in the healthcare sector. They were recruited among individuals attending dermatologic consultations in our service, companions of patients seen for other dermatoses, adults enrolled in professional courses at a nearby school, and their relatives, as well as community contacts from local churches.

Patients answered a clinical and demographic questionnaire and the EMIC-AP stigma scale. Contacts and the GP group completed a sociodemographic questionnaire and the EMIC-CSS scale. Both instruments were applied in their validated Brazilian versions.^18^, ^19^

### Data collection

Data collection was automated through an online electronic form (Google Forms), anonymous, and protected by encryption. The collected data were tabulated using Microsoft Excel 2013.

### Statistical analysis

For descriptive statistics, categorical and ordinal variables were expressed as percentages, and quantitative variables as mean and standard deviation, or as median and interquartile range (P25–P75) when normality was not indicated by the Shapiro-Wilk test.^20^

Proportions were compared using the Chi-Square test or the Chi-Square test for trend, supplemented by residual analysis of the contingency table. The mean age among groups was compared using ANOVA with Šidák *post hoc* analysis.^21^, ^22^

Terms cited in the open-ended question were processed with WordArt (http://www.wordart.com/create) to generate word clouds containing up to 80 of the most frequent words.^23^

The correlation among items within each group was explored using network analysis with the EBICglasso estimator.^24^

The variation of stigma scores in each group was tested against each clinical-demographic covariate using a generalized linear model with robust analysis, adjusted to the underlying probability distribution. Covariates with p < 0.20 were included in the final multivariate model.^25^

The effect size was estimated by the regression beta coefficient and its Standard Error (SE).

The internal consistency of the questionnaires was evaluated using McDonald’s ω coefficient, considered adequate when > 0.7.^26^

Stigma scores for each individual in the three groups were standardized (θvalue) using item response theory (Samejima’s graded response model) and compared across groups using a generalized linear model adjusted for gender, age, education, and religious practice.^27^

Data were analyzed using IBM SPSS 31v and JASP 0.95. A p-value < 0.05 was considered statistically significant.^28^

### Sample size calculation

The sample size was defined pragmatically according to the feasibility of recruitment in the study period and to ensure adequate psychometric and multivariate precision. For the reliability and dimensionality analyses of the EMIC-AP and EMIC-CSS scales, a minimum of five participants per item was considered sufficient to estimate internal consistency with a 95% Confidence Interval width below 0.10, resulting in a minimum sample of 75 individuals per group.

## Results

A total of 214 patients, 104 contacts, and 393 GPs were analyzed after the exclusion of one patient and two GP participants due to incomplete data.

The main sociodemographic characteristics of the groups are shown in Table 1. A higher proportion of males was observed among patients, whereas females predominated among contacts. Patients were older than participants in the other groups. Patients had lower educational levels, whereas the GP group showed the highest. In all groups, more than 80% identified themselves as Christians.

**Table 1:** Sociodemographic data of patients, contacts, and the general population not related to the disease (GP).

Variables	Patients	Contacts	GP	p-value
**N**	214	104	393	‒
**Age (years), mean (SD)**	51 (15)^a^	42 (14)	43 (15)	**<0.01**
**Gender, n (%)**				**<0.01**
Male	130 (61%)^a^	24 (23%)^b^	208 (53%)	
Female	84 (39%)	80 (77%)	184 (47%)	
Preferred not to disclose	- (-)	- (-)	1 (0%)	
**Education, n (%)**				**<0.01**
No formal education	16 (8%)^a^	3 (3%)	3 (1%)^b^	
Elementary	100 (47%)	29 (28%)	40 (10%)	
High School	81 (38%)	58 (56%)^a^	142 (36%)	
University education	17(8%)^b^	14 (14%)^b^	208 (53%)^a^	
**Religion, n (%)**				0.43
Christian	188 (88%)	90 (86%)	290 (73%)	
Jewish	- (-)	- (-)	3 (1%)	
Afro-Brazilian	1 (1%)	- (-)	1 (0%)	
Spiritist	1 (1%)	- (-)	8 (2%)	
No religion	23 (11%)	14 (14%)	44 (11%)	
Preferred not to disclose / Other	- (-)	- (-)	47 (12%)	
**Religious practice, n (%)**				**<0.01**
Yes	102 (48%)	41 (39%)^b^	226 (57%)^a^	
Somewhat	45 (21%)	26 (25%)	81 (21%)	
No	67 (31%)	37 (36%)^a^	85 (22%)^b^	
**Know what leprosy is, n (%)**	198 (93%)^a^	93 (89%)	333 (85%)^b^	**0.02**
**Leprosy affects, n (%)**				**<0.01**
Skin	88 (41%)^b^	58 (45%)^b^	343 (81%)^a^	
Bones	19 (9%)^a^	17 (13%)	12 (3%)^b^	
Nerves	97 (45%)^a^	48 (38%)^a^	48 (11%)^b^	
Other / Don’t know	12 (6%)	5 (4%)	21 (5%)	

SD, Standard Deviation; GP, General Population not related to the disease.

a Higher-than-expected value.

b Lower-than-expected value.

Regarding knowledge about leprosy, over 80% of participants reported knowing what the disease is, though this was less frequently stated among GP individuals. Concerning the affected organ/system, 81% of GP participants reported that leprosy affects the skin, whereas neurological involvement was more frequently recognized by patients and contacts (Table 1). The GP group reported practicing religion more often, although only 56% recognized leprosy as endemic in the country.

At the time of the interview, most patients presented visible physical deformities (58%), and 60% were classified with grade 2 physical disability (G2D). Additionally, 40% of the cases corresponded to newly diagnosed patients.

The stigma-related questionnaires demonstrated high internal consistency (ω-McDonald, 95% CI): EMIC-AP = 0.75 (0.70–0.80) and EMIC-CSS = 0.83 (0.81–0.85).

Among patients, the mean (SD) EMIC-AP score was 16 (9), ranging from 0 to 39. Table 2 presents the frequency of responses for each EMIC-AP item. The highest scores were observed for items 1 (preferred that others did not know about the disease), 4 (shame), and 14 (social withdrawal). The lowest scores were recorded for items 2 (talking with someone close), 6 (contagion of close persons), 12 (difficulty with a family member’s marriage), and 13 (work exclusion).

**Table 2:** EMIC-AP item scores – Patients (n = 214).

Items	Yes	Possibly	Uncertain	No
1. If possible, would you prefer to keep people from knowing about your leprosy?	108 (51%)	3 (1%)	1 (1%)	102 (48%)
2. Have you discussed your leprosy with the person you consider closest to you, the one whom you usually feel you can talk to most easily?^a^	180 (84%)	2 (1%)	- (-)	32 (15%)
3. Do you think less of yourself because of your leprosy? Has it reduced your pride or self-respect?	83 (39%)	8 (4%)	2 (1%)	121 (57%)
4. Have you ever been made to feel ashamed or embarrassed because of your leprosy?	125 (58%)	5 (2%)	- (-)	84 (39%)
5. Do your neighbours, colleagues or others in your community have less respect for you because of your leprosy?	55 (26%)	15 (7%)	19 (9%)	125 (58%)
6. Do you think that contact with you might have any bad effects on others around you even after you have been treated?	40 (19)	6 (3%)	12 (6%)	156 (73%)
7. Do you feel others have avoided you because of your leprosy?	83 (39%)	8 (4%)	7 (3%)	116 (54%)
8. Would some people refuse to visit your home because of this condition even after you have been treated?	81 (38%)	17 (8%)	4 (2%)	112 (52%)
9. If they knew about it would your neighbours, colleagues or others in your community think less of your family because of your leprosy?	75 (35%)	9 (4%)	15 (7%)	115 (54%)
10. Do you feel that your leprosy might cause social problems for your children in the community?	65 (30%)	6 (3%)	5 (2%)	138 (65%)
11A. Do you feel that this disease might make it difficult for you to marry? (unmarried)	24 (23%)	8 (8%)	5 (5%)	69 (65%)
11B. Do you feel that this disease has caused problems in your marriage? (married)	27 (22%)	4 (3%)	3 (2%)	89 (72%)
12. Do you feel that your leprosy makes it difficult for someone else in your family to marry?	28 (13%)	2 (1%)	8 (4%)	176 (82%)
13. Have you been asked to stay away from work or social groups?	40 (19%)	3 (1%)	- (-)	171 (80%)
14. Have you decided on your own to stay away from work or social group?	112 (52%)	2 (1%)	- (-)	100 (47%)
15. Because of your leprosy, do people think you also have other health problems?	85 (40%)	8 (4%)	12 (6%)	109 (51%)

a Inverted item.

**Figure 1:**
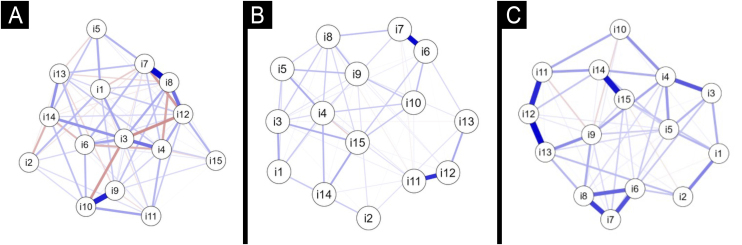
Network structures of stigma-related items across the three study groups. (A) EMIC-AP for patients. (B) EMIC-CSS for contacts. (C) EMIC-CSS for the General Population (GP). Each node represents one item of the corresponding scale, and edges represent partial correlations between items. Thicker edges indicate stronger conditional associations. Node centrality reflects its contribution to the variability of stigma scores within the scale. Items with closer proximity and thicker connecting edges form conceptual clusters with similar behavior.

Fig. 1A presents the EMIC-AP network diagram, indicating that items 3 (negative self-opinion), 4 (shame), and 6 (contagion of close persons) were the most representative of stigma variation among patients. Items 7 (avoidance by others) and 8 (visiting one’s home) showed a strong correlation, as did items 9 (negative opinion of colleagues) and 10 (social repercussions for children), suggesting parallel conceptual domains in these pairs of items.

When exploring clinical and sociodemographic factors associated with stigma scores among patients (Table 3), higher EMIC-AP scores were observed among female participants.

**Table 3:** Associations between clinical and demographic data and EMIC-AP scores among patients (n = 214).

Variables	Bivariate analysis		Multivariate analysis^a^	
	Coefficient β (SE)	p-value	Coefficient β (SE)	p-value
Male gender	−3.3 (1.3)	***0.01***	−2.9 (1.3)	***0.03***
University education	−1.8 (1.7)	0.30	‒	‒
Age > 45-years	0.9 (1.4)	0.51	‒	‒
Jewish/Christian religion	0.0 (1.9)	0.99	‒	‒
Religious practice	2.5 (1.3)	0.05	2.0 (1.3)	0.11
Disability G2D	0.57 (1.3)	0.67	‒	‒
Deformity	−0.21 (1.3)	0.88	‒	‒

SE, Standard Error.

a Multivariate analysis including covariates with p ≤ 0.20 in the bivariate analysis.

Among contacts, the mean (SD) EMIC-CSS score was 15 (7), ranging from 0 to 26. Table 4 shows the frequency of responses for each EMIC-CSS item. The highest scores were found for items 4 (negative opinion), 14 (difficulty finding a job), and 15 (buying food). The lowest scores were recorded for items 2 (negative opinion about oneself) and 13 (relative’s marriage).

**Table 4:** EMIC-CSS item scores – Contacts (n = 104).

Items	Yes	Possibly	Uncertain / No
1. A person with leprosy would try to keep others from knowing, if possible.	71 (68%)	2 (2%)	31 (30%)
2. If a member of your family had leprosy, you would think less of yourself because of that person’s problem.	23 (22%)	3 (3%)	78 (75%)
3. In your community, leprosy causes shame or embarrassment.	64 (62%)	6 (6%)	34 (33%)
4. Other people might think less of a person with leprosy.	81 (78%)	9 (9%)	14 (14%)
5. Knowing that someone has leprosy would have a bad effect on other people.	60 (60%)	8 (8%)	36 (35%)
6. Other people in your community would avoid a person who has leprosy.	15 (14%)	49 (47%)	40 (39%)
7. Other people would refuse to visit the home of someone with leprosy.	12 (12%)	50 (48%)	42 (40%)
8. People in your community would think less of the family of a person with leprosy.	52 (50%)	10 (10%)	42 (40%)
9. Leprosy would cause problems for a person’s family.	42 (40%)	14 (14)	48 (46%)
10. A family would be worried about telling others that one of them had leprosy.	55 (53%)	4 (4%)	45 (43%)
11. Leprosy would be a problem for a person to get married.	30 (29%)	8 (8%)	66 (64%)
12. Leprosy would cause problems in a person’s marriage.	29 (28%)	7 (7%)	68 (65%)
13. Having leprosy would cause problems for a relative of that person to get married.	17 (16%)	4 (4%)	83 (80%)
14. Having leprosy would make it difficult for a person to find work.	74 (71%)	9 (9%)	21 (20%)
15. People would not like to buy food from someone who has leprosy.	77 (74%)	7 (7%)	20 (19%)

Fig. 1B presents the EMIC-CSS network diagram for contacts, indicating that items 15 (buying food) and 10 (family disclosure of the disease) were the most representative of stigma variation among this group. Items 6 (avoidance) and 7 (visiting the home), as well as 12 (relationship problems) and 11 (difficulty marrying), were strongly correlated, suggesting conceptual proximity between these pairs.

When analyzing sociodemographic factors associated with stigma scores among contacts (Table 5), higher EMIC-CSS scores were observed among individuals with higher educational levels.

**Table 5:** Associations between sociodemographic data and EMIC-CSS scores among contacts (n = 104).

Variables	Bivariate analysis		Multivariate analysis^a^	
	Coefficient β (SE)	p-value	Coefficient β (SE)	p-value
Male gender	−0.5 (1.6)	*0.73*	‒	‒
University education	2.8 (1.5)	0.06	3.2 (1.4)	*0.03*
Age > 45-years	−2.4 (1.3)	0.07	−2.1 (1.3)	0.11
Jewish/Christian religion	1.6 (1.4)	0.26	‒	‒
Religious practice	1.8 (1.3)	0.17	1.7 (1.3)	0.19

SE, Standard Error.

a Multivariate analysis including covariates with p ≤ 0.20 in the bivariate analysis.

For the GP group, the mean (SD) EMIC-CSS score was 12 (6), ranging from 0 to 28. Table 6 presents the frequency of responses for each EMIC-CSS item. The highest scores were found for items 4 (negative opinion) and 15 (relative’s marriage), while the lowest were for items 2 (negative opinion about oneself) and 11 (difficulty marrying).

**Table 6:** Scores of EMIC-CSS items – GP (n = 393).

Items	Yes	Possibly	Uncertain / No
1. A person with leprosy would try to keep others from knowing, if possible.	162 (41%)	138 (35%)	93 (24%)
2. If a member of your family had leprosy, you would think less of yourself because of that person’s problem.	21 (5%)	25 (6%)	347 (88%)
3. In your community, leprosy causes shame or embarrassment.	116 (30%)	114 (29%)	163 (42%)
4. Other people might think less of a person with leprosy.	186 (47%)	137 (35%)	70 (18%)
5. Knowing that someone has leprosy would have a bad effect on other people.	101 (26%)	153 (39%)	139 (35%)
6. Other people in your community would avoid a person who has leprosy.	116 (30%)	143 (36%)	134 (34%)
7. Other people would refuse to visit the home of someone with leprosy.	100 (25%)	162 (41%)	131 (33%)
8. People in your community would think less of the family of a person with leprosy.	79 (20%)	141 (36%)	173 (44%)
9. Leprosy would cause problems for a person’s family.	85 (22%)	131 (33%)	177 (45%)
10. A family would be worried about telling others that one of them had leprosy.	73 (19%)	150 (38%)	170 (43%)
11. Leprosy would be a problem for a person to get married.	30 (8%)	124 (32%)	239 (61%)
12. Leprosy would cause problems in a person’s marriage.	53 (14%)	129 (33%)	211 (54%)
13. Having leprosy would cause problems for a relative of that person to get married.	41 (10%)	89 (23%)	263 (67%)
14. Having leprosy would make it difficult for a person to find work.	145 (37%)	141 (36%)	107 (27%)
15. People would not like to buy food from someone who has leprosy.	153 (39%)	127 (32%)	113 (29%)

Fig. 1C shows the EMIC-CSS network diagram for the GP group, indicating that items 15 (buying food) and 9 (family problems) were the most representative of stigma variation in the population of this endemic area. Items 14 (difficulty finding a job) and 15, 6 (avoidance), 7 (visiting the home), and 8 (negative family opinion); 12 (relationship problems) and 11 (difficulty marrying); as well as 12 and 13 (relatives’ marriage) showed strong inter-item correlations, suggesting conceptual overlap among these domains.

When exploring sociodemographic factors associated with stigma scores among the GP (Table 7), higher EMIC-CSS scores were observed among younger individuals and among those who practiced religion.

**Table 7:** Associations between sociodemographic data and EMIC-CSS scores among the GP (n = 393).

Variables	Bivariate analysis		Multivariate analysis^a^	
	Coefficient β (SE)	p-value	Coefficient β (SE)	p-value
Male gender	0.6 (0.6)	0.31	‒	‒
University education	0.0 (0.6)	0.94	‒	‒
Age > 45-years	−1.6 (0.6)	*0.01*	−1.3 (0.6)	*0.03*
Jewish/Christian religion	1.6 (0.8)	0.05	1.1 (0.9)	0.27
Religious practice	1.6 (0.6)	*0.01*	1.3 (0.7)	*0.04*

SE, Standard Error.

a Multivariate analysis including covariates with p ≤ 0.20 in the bivariate analysis.

The EMIC scores of each individual from the three groups were standardized (θ-values) using an Item Response Theory model. When adjusted for gender, age-group, education, Jewish-Christian religion, and religious practice, the mean (SD) standardized θ values for contacts and GP were higher (p < 0.05) than for patients: 0.02 (0.91) vs. -0.06 (0.92) vs. -0.26 (0.91), respectively. This multivariable approach showed that only religious practice was significantly associated with higher standardized stigma scores (β = 0.21; p < 0.01) in all groups, but not gender, age-group, or education (Supplementary Table 1).

Regarding the open question, “What comes to mind when you hear about leprosy?”, there was some overlap in the most frequent words among patients and contacts (disease, cure, treatment, prejudice, skin), while among GP participants, terms such as leprosy, spot, wound, and contagious emerged (Supplementary Fig. 1).

## Discussion

In this study, different aspects of leprosy-related stigma were explored across three independent groups: patients, contacts, and GPs from an endemic region of the Amazonian region. The persistence of stigma surrounding leprosy, even in a context of curative treatment and long-standing public policies, was evident across all study groups, which still display attitudes and beliefs that reflect fear of contagion, shame, and social exclusion, confirming that the symbolic burden of leprosy remains a relevant social determinant of its control in endemic regions.

The mean EMIC-AP score among patients in this study is aligned with other Brazilian series that report scores between 14 and 18.^18^, ^29^ Higher responses to items concerning concealment, shame, and social withdrawal demonstrate that emotional and behavioral responses to the disease continue to influence self-perception and social interactions. Although most participants recognized leprosy as curable, self-stigmatization persists, showing that the psychosocial impact extends beyond the effective medical treatment.

Network analysis highlighted the centrality of items expressing negative self-opinion and perceived avoidance by others, highlighting the interaction between internalized and enacted stigma. The proximity of domains related to social exclusion (work, marriage, and family relations) suggests that the persistence of stigma is anchored in everyday social roles, not only in fear of contagion. These results are consistent with contemporary stigma models that emphasize its multidimensional and relational nature.^30^

The paradoxical finding of higher standardized stigma scores among contacts and the general population than among patients reveals that direct experience with the disease may mitigate prejudice. While affected individuals tend to reinterpret their condition through treatment and professional support, contacts often reproduce collective fears and misconceptions. This gradient reinforces the importance of community education and the inclusion of family members in counseling and follow-up programs.

In Brazil, the “National Strategy for Confronting Leprosy 2024–2030 (Portuguese: Plano Nacional de Enfrentamento da Hanseníase 2023–2030, PNHansen)” reinforces the strategic alignment with the WHO Global Leprosy Strategy by prioritizing intersectoral actions that integrate health, education, and social protection. The plan emphasizes stigma reduction through community education campaigns, inclusion of leprosy content in school curricula, and active participation of affected persons as advocates for awareness. It also promotes psychosocial rehabilitation, vocational reintegration, and the expansion of self-care and peer-support groups within primary health care networks. Communication initiatives target misinformation and moral prejudice, aiming to reframe public perceptions of leprosy as a curable and socially manageable disease. These combined measures are essential to advance toward the national goal of “Zero Disabilities and Zero Stigma” by 2030.^31^

In the GP group, the persistence of negative perceptions, particularly regarding employability and marriage, demonstrates that social discrimination is not restricted to traditional or low-income contexts. The symbolic association between leprosy and moral impurity or physical deformity continues to shape public attitudes, despite decades of biomedical progress.

These findings support the literature emphasizing that leprosy-related stigma can act as one barrier to treatment adherence and disease control, while also negatively affecting case detection and the social support provided. Items that assess the belief that society judges people with leprosy negatively revealed a strong perception of social stigma among contacts. This anticipation of judgment fosters silence and social withdrawal, intensifying psychological distress and hindering the search for support and treatment. Moreover, recognizing the existence of such prejudice within the social environment may lead to emotional distancing and even neglect of the patient’s needs, extending the consequences of stigma beyond the affected individual. Community interventions based on media campaigns have the potential to gradually educate the population on leprosy and reduce stigma.^31^, ^32^, ^33^ Surveys conducted in India have observed low or inadequate levels of knowledge about leprosy among people affected by the disease. Limited understanding of leprosy has been associated with greater social distancing, negative attitudes toward affected individuals, and poor treatment adherence.^34^

In a study conducted with 135 participants in Nepal, the EMIC-AP scale was used to assess the level of perceived stigma and identify the risk factors contributing to it. The findings showed that several factors shape the perception of stigma among people affected by leprosy. In this sample, 63% were male, and 79% identified as Hindu. Higher levels of perceived stigma were significantly associated with individuals who had physical disabilities resulting from the disease; approximately 51% of participants presented grade 2 disability, as well as with low socioeconomic status and low educational attainment (55% were illiterate). In the same study, 71% reported lacking adequate knowledge about the disease, and 80% perceived it as severe and difficult to treat; these factors were also associated with higher levels of stigma among affected individuals.^35^

The EMIC-AP was administered to 220 participants from Brazil. Three variables were identified as associated with perceived stigma: social class, physical disability resulting from leprosy, and low knowledge about the disease. Another important finding was the relationship between economic income and increased perception of stigma, reflecting the persistence of social stereotypes that associate poverty with stigmatized health conditions. Individuals affected by leprosy with lower economic status reported significantly higher levels of perceived stigma compared to those with higher income, indicating a strong link between poor living conditions and stigma intensity. In addition to these factors, visible deformities, physical limitations, and fear of contagion were identified as reasons related to unemployment among people with the disease, with substantial repercussions for their families and communities.^36^

Female patients presented higher stigma scores, as observed in other studies.^37^, ^38^ This pattern may reflect gendered expectations regarding aesthetics, self-image, and social acceptance, reinforcing the need for psychosocial support tailored to women. The high prevalence of visible deformities and grade 2 disabilities amplifies this effect, illustrating the bidirectional relationship between physical and social impairment.

Among contacts, a higher educational level correlated with greater stigma, an unexpected finding suggesting that knowledge alone does not guarantee attitudinal change. Without lived experience or direct dialogue, information may reinforce perceived risk rather than empathy. In the GP group, younger age and active religious practice were associated with higher stigma scores, possibly linked to moral or symbolic interpretations of disease causality.

The greater stigma observed among non-affected individuals highlights the need to broaden health communication strategies beyond the clinical environment. Campaigns should emphasize the low transmissibility of treated cases, the reversibility of the disease, and the rights of cured individuals, while actively involving community leaders and religious institutions. These findings are similar to those of a study conducted in New Zealand, where more than 50% of respondents to the EMIC-CSS believed that leprosy would cause shame or embarrassment, social isolation, family difficulties, and problems in finding employment.^39^ The items related to negative opinion (item 4), avoidance (item 6), refusal to visit the person’s home (item 7), family concern about disclosing the disease (item 10), and work-related difficulties (items 14 and 15) were those that contributed most to higher scores on the scale.

Reducing stigma requires intersectoral action that integrates education, social protection, and rehabilitation policies. Community-based approaches that promote contact and dialogue with affected persons have shown measurable impact on prejudice reduction. Psychosocial rehabilitation, professional reintegration, and positive media representation should complement biomedical interventions.^32^

Between 2002 and 2005, the Stigma Elimination Programme (STEP) was developed in Nepal to address perceived, internalized, and enacted stigma by transforming the image of people affected by leprosy from “victims” into agents of change through an emphasis on empowerment. In this initiative, individuals affected by leprosy were trained to serve as facilitators in self-care groups within their villages. As these groups became more cohesive, they evolved into self-help groups that established cooperatives and microenterprises. Over time, they began to include other socially excluded individuals, generating benefits for their communities and gaining local recognition. The impact of STEP was evaluated through a participatory approach involving community representatives, group members, and program facilitators. Additionally, the Social Participation Scale (P-Scale) was applied. It demonstrated greater community participation among formerly stigmatized individuals in the areas where these groups were established compared to those where the program was not implemented.^40^

This study has some limitations regarding using a non-randomized sample. The cross-sectional design does not allow causal inference, and data collection from a single reference center may limit representativeness. However, the inclusion of three sampling strata and the use of robust adjusted modeling strengthen the validity and generalizability of the findings, which should be confirmed along with other regions of the country. Despite assessing disability, this study did not consider the effect of leonine face and multiple visible lesions as factors associated with stigma. Future longitudinal studies should also explore temporal changes in stigma perception after treatment initiation and community interventions. The assessment of the psychometric performance of these instruments, as well as the categorization of their scores, is warranted.

The GP group does not fully represent the Brazilian population, particularly with regard to educational attainment. Higher educational level may indeed influence stigma perception. Although greater education is often associated with improved health literacy, our findings showed that higher schooling among contacts, and to a lesser extent among GP participants, was paradoxically associated with higher stigma scores. This suggests that education alone does not eliminate misconceptions or social distancing related to leprosy.

The persistence of social stigma, despite effective cure and widespread information, emphasizes the need to view leprosy not only as an infectious disease but as a social condition marked by exclusion and silence. Overcoming stigma demands continuous, community-based dialogue capable of transforming fear and prejudice into solidarity, an indispensable step toward achieving the “zero stigma” target of the Global Leprosy Strategy.^41^

Leprosy-related stigma remains a persistent and multifaceted barrier to disease control and social inclusion, even in the era of curative treatment. The persistence of shame, concealment, and discriminatory attitudes highlights that biomedical advances alone are insufficient to eliminate the social consequences of the disease. To achieve the goals of the WHO “Zero Leprosy” strategy, interventions must go beyond pharmacological cure and prioritize education, social reintegration, and community engagement. Integrating psychosocial care, public awareness campaigns, and intersectoral policies can foster empathy, dismantle prejudice, and promote full citizenship for people affected by leprosy. In endemic regions such as the Brazilian Amazon, confronting stigma is not merely an ethical imperative but an essential step toward true elimination of the disease and its enduring legacy of exclusion.^17^

These findings highlight the need for targeted public health strategies in the Amazonian region, where geographic isolation and social vulnerability sustain leprosy transmission and stigma. Strengthening community-based surveillance, psychosocial support, and health education, especially through primary care and community health workers, could promote early detection and social reintegration. Intersectoral actions integrating health, education, and social protection, as advocated by the PNHansen, are essential to address the social determinants underlying the persistence of leprosy in the Brazilian Amazon.

## Conclusion

This study demonstrated that leprosy-related stigma is not confined to those directly affected by the disease but extends to contacts and the broader community, reflecting deeply rooted cultural misconceptions and social distance.

## ORCID ID

Patrícia Tavares Cruz: 0009-0009-6816-585X

Carolina Talhari: 0000-0003-2283-069X

Valderiza Lourenço Pedrosa: 0000-0002-9169-6116

Raquel da Mata Serique: 0009-0006-9625-732X

Amanda Gabrielle dos Santos Cordeiro: 0000-0002-5245-0594

Jaqueline da Silva Mendes: 0009-0003-5586-907X

Susan Smith Doria: 0000-0002-4431-7099

Jorge Ewerton dos Santos Sales: 0009-0008-9553-166X

Alexandra de Freitas Costa: 0009-0009-3105-9682

Sinésio Talhari: 0000-0001-9753-6706

## Research data availability

The entire dataset supporting the results of this study was published in this article.

## Financial support

This study was funded by FAPEAM(Fundação de Amparo à Pesquisa do Estado do Amazonas, Brazil) through the “Programa de Apoio à Formação em Ciências Dermatológicas – PRODERM-RH” (grant nº 010/2023). HAM, FAL, and ST are recipients of FAPEAM PVN-II research fellowships. PTC received funding from FAPEAM (grant:POSGRAD 002/2023).

## Authors' contributions

Patrícia Cruz: Study conception and design; data collection; data analysis and interpretation; manuscript drafting; critical literature review; critical manuscript revision; approval of the final version of the manuscript.

Sinésio Talhari: Study conception; Manuscript drafting; critical literature review; critical manuscript revision; approval of the final version of the manuscript.

Carolina Talhari: Manuscript drafting; Critical literature review; critical manuscript revision; approval of the final version of the manuscript.

Valderiza Lourenço Pedrosa: Review of data analysis and interpretation; statistical analysis; critical literature review; critical manuscript revision.

Hélio Miot: Study conception and design; data analysis and interpretation; statistical analysis; manuscript drafting; critical literature review; critical manuscript revision; approval of the final version of the manuscript.

Alexandra Costa: Data collection; Data interpretation; critical literature review; critical manuscript revision; approval of the final version of the manuscript.

Jorge Sales: Data collection; Data interpretation; critical literature review; critical manuscript revision; approval of the final version of the manuscript.

Susan Doria: Data collection; data interpretation; critical literature review; critical manuscript revision; approval of the final version of the manuscript.

Raquel Serique: Data collection; Data interpretation; critical literature review; critical manuscript revision; approval of the final version of the manuscript.

Amanda Cordeiro: Data collection; data interpretation; critical literature review; critical manuscript revision; approval of the final version of the manuscript.

Jaqueline da Silva Mendes: Data collection; data interpretation; critical literature review; critical manuscript revision; approval of the final version of the manuscript.

## Conflicts of interest

None declared.
