# Supplementary material for: Stigma associated with leprosy among patients, contacts, and the general population in an endemic region of Brazil
Source: An Bras Dermatol. 2026 Mar 9;101(2):501301. doi: 10.1016/j.abd.2026.501301 (PMC12993181; doi:10.1016/j.abd.2026.501301)

**ABD-D-25-00753**

**Supplementary Material**

**Supplementary Table 1** Multivariate analysis of the standardized (θ-values) EMIC scores from the three groups, adjusted for gender, age-group, education, Jewish-Christian religion, and religious practice.

| **Variables** | **Coefficient β (SE)** | **p-value** |
| --- | --- | --- |
| Group |  |  |
| *General Population* | 0.18 (0.09) | **0.039** |
| *Contacts* | 0.27 (0.11) | 0.019 |
| *Patients* | (-) |  |
| Male gender | -0.02 (0.07) | 0.769 |
| University education | 0.02 (0.08) | 0.784 |
| Age (> 45-years) | -0.07 (0.07) | 0.316 |
| Jewish/Christian religion | 0.03 (0.10) | 0.767 |
| Religion practice | 0.21 (0.08) | **0.005** |

P (model) = 0.004.

**Supplementary Figure 1** Word clouds related to the question “What comes to mind when leprosy is mentioned?” (A) Patients, (B) Contacts, and (C) GP. Results in Portuguese are more reliable in relation to the (Brazilian) participant’s discourse.


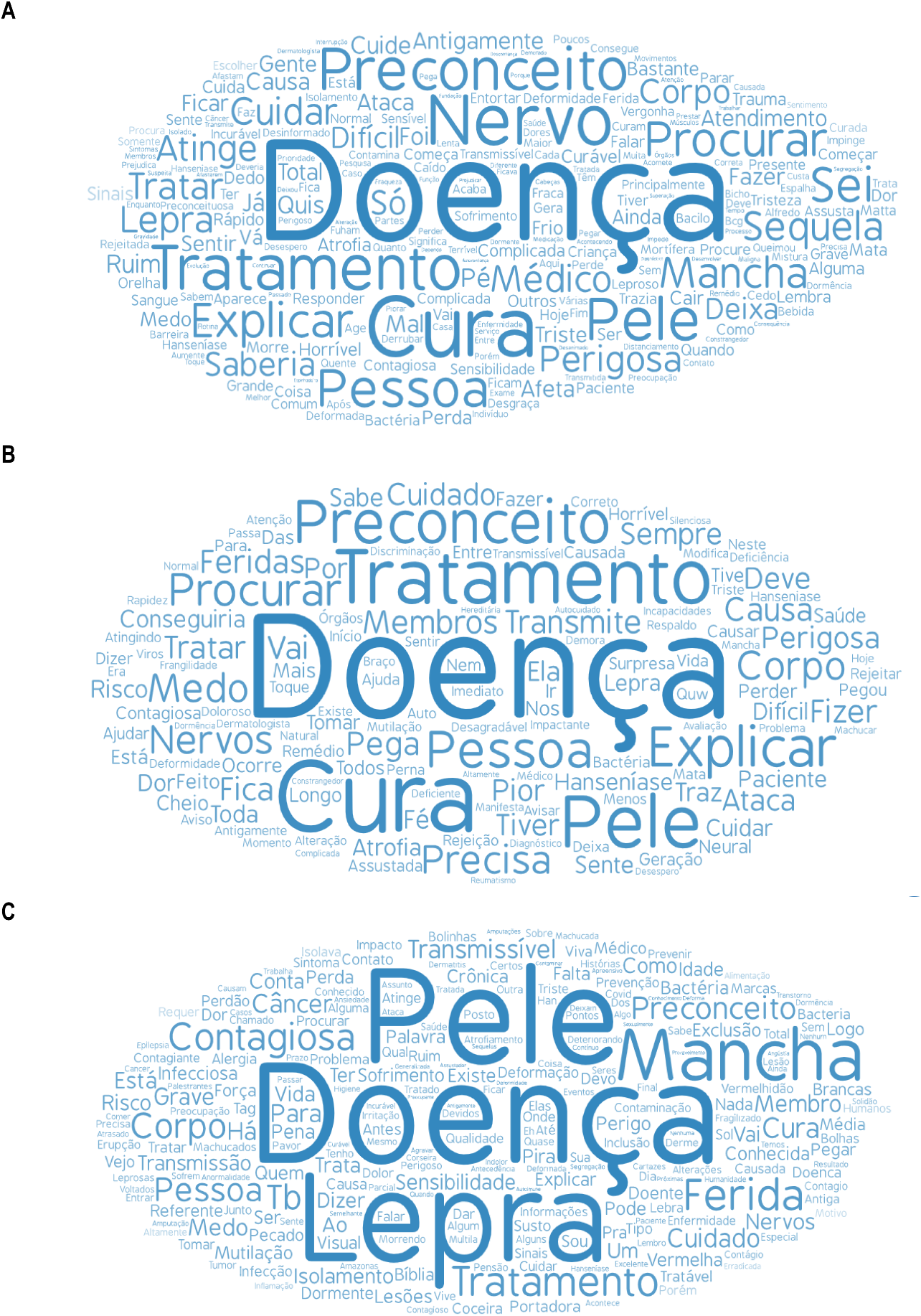

Supplement: Supplementary file 1 [file mmc1.docx]
